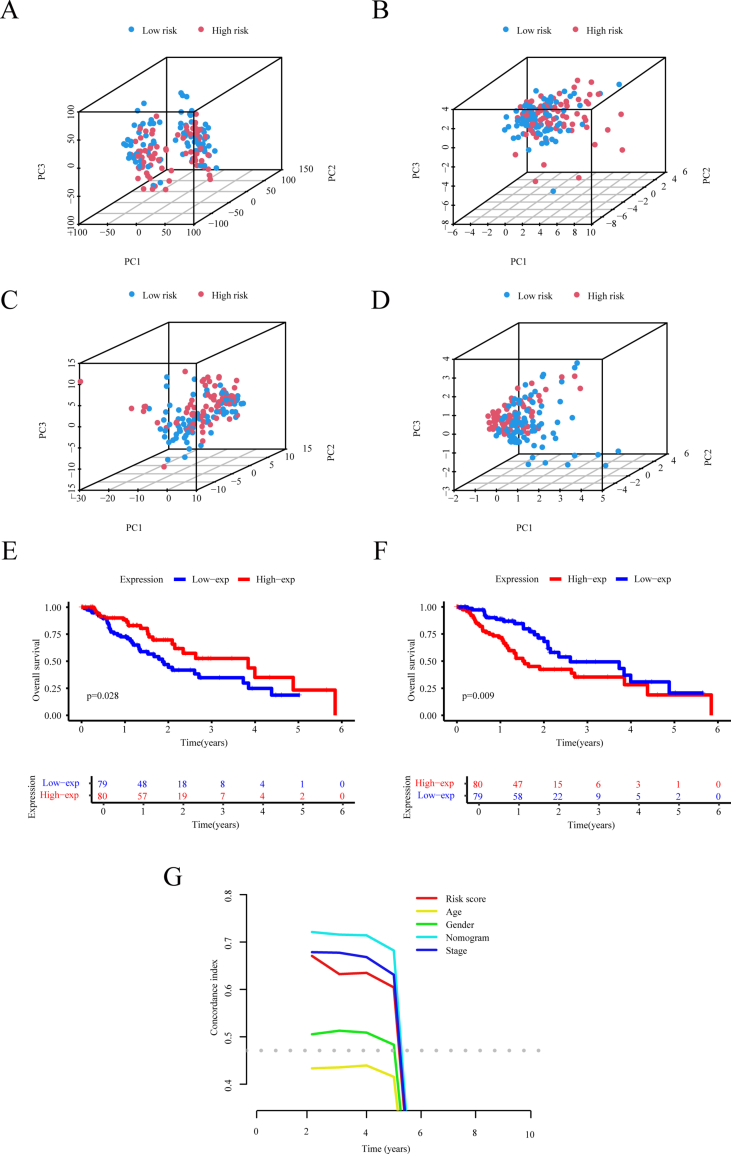# Identification of a novel prognostic cuproptosis-associated LncRNA signature for predicting prognosis and immunotherapy response in patients with esophageal cancer

**DOI:** 10.1016/j.heliyon.2024.e30277

**Published:** 2024-04-26

**Authors:** Xinhai Sun, Liming Li, Xiaojie Yang, Dan Ke, Qihong Zhong, Yuanchang Zhu, Litao Yang, Zhenyang Zhang, Jiangbo Lin

**Affiliations:** a Department of Thoracic Surgery, Fujian Institute of Thoracic and Cardiac Surgery, Fujian Medical University Union Hospital, Fuzhou, China; b Department of Thoracic Surgery, The Third Affiliated Hospital of Chongqing Medical University, Chongqing, China; c Heilongjiang Key Laboratory of tissue damage and repair, College of life sciences, Mudanjiang Medical University, Mudanjiang, China; d Department of Colorectal Surgery, Fujian Medical University Union Hospital, Fuzhou, China; e Department of Thoracic Surgery, Baoji Traditional Chinese Medicine Hospital, Shaanxi, China

**Keywords:** Esophageal cancer, Cuproptosis, lncRNA, Prognostic signature, *UGDH-AS1*

## Abstract

Nowadays, effective prognostic models for esophageal cancer (ESCA) are still lacking. Long noncoding RNAs (lncRNAs) are commonly utilized as indicators for diagnosing cancer and forecasting patient outcomes. Cuproptosis is regulated by multiple genes and is crucial to the progression of ESCA. However, it is not yet clear what role the cuproptosis-associated lncRNAs (CuALs) play in ESCA. To tackle this problem, a prognostic signature incorporating three CuALs was created. This signature was constructed by the use of the least absolute shrinkage and selection operator (LASSO) and multivariate Cox regression. Subsequently, the signature effectively stratified ESCA samples into a high-risk group and a low-risk group. Those in the low-risk group demonstrated extended overall survival (OS), as well as increased infiltration of T cells, macrophages, and NK cells, suggesting a potentially enhanced response to immunotherapy. The ROC curve analysis demonstrated that this prognostic signature outperformed conventional clinical factors in predicting patient prognosis (AUC = 0.708). K-M survival analysis and correlation analysis identified *UGDH-AS1* (a CuAL) as a protective factor positively associated with patient prognosis. The results of RT-qPCR and wound healing assays indicated that *UGDH-AS1* is overexpressed in ESCA and could inhibit cancer cell migration. In general, the prognostic signature of CuALs demonstrated a robust capability in forecasting the immune environment and patient prognosis, highlighting its potential as a tool for enhancing personalized treatment strategies in ESCA.

## Highlights

• The prognostic signature of cuproptosis-associated lncRNAs can accurately and independently predict patient prognosis.
• Immunotherapy may be more effective for low-risk ESCA patients in the prognostic signature.
• *UGDH-AS1*, a cuproptosis-associated lncRNA, is upregulated in ESCA and associated with improved patient survival.

## 1 Introduction

Globally, esophageal cancer (ESCA) stands as the sixth leading oncological reason for mortality [1]. Squamous cell cancer accounts for approximately 85 % of ESCA cases [2]. Current treatment strategies for ESCA vary based on the pathological state and cancer subtype. Locally advanced patients may benefit from chemotherapy, targeted therapy, and postsurgical resection chemotherapy [3,4]. In cases of metastasis, chemotherapy and nonsurgical interventions are typically recommended [5]. Unfortunately, owing to the absence of accurate methods for early detection, most individuals are diagnosed with ESCA at an advanced stage, which greatly diminishes their 5-year survival prospects [6]. Recently, immunotherapy, especially immune checkpoint inhibitors (ICIs), has emerged as a preferred treatment option for non-small cell lung cancer and malignant melanoma [5]. Notably, ICIs, either alone or in combination with chemoradiotherapy, have progressively evolved into first-line treatments for ESCA and have shown remarkable therapeutic efficacy [7,8]. However, it is crucial to acknowledge that responses to ICIs can vary significantly among individuals. Consequently, it is urgently needed to construct a prognostic signature that accurately forecasts outcomes for individuals with ESCA and identifies those responsive to immunotherapy.

Numerous studies have demonstrated that cell death mechanisms, such as apoptosis [9], ferroptosis [10], and necroptosis [11], have an impact on the course and progression of ESCA. Tsvetkov et al. have confirmed that cuproptosis, a recently identified cell death mechanism, is strongly associated with mitochondria [12]. The basic mechanism is copper's interaction with the components of the lipidized tricarboxylic acid cycle. This results in the excessive accumulation of fatty acylated proteins, which in turn triggers the activation of the proteotoxic stress response and causes cell death. Furthermore, research has shown that a prognostic signature utilizing six genes related to copper metabolism can effectively distinguish hepatocellular carcinoma patients into subgroups with different prognoses [13]. What's more, prognostic signatures using cuproptosis-related genes have shown success in predicting potential drugs for treating ovarian cancer [14]. However, the impact of cuproptosis on ESCA progression remains unclear.

Long noncoding RNAs (lncRNAs) are essential for regulating immune responses [15]. LncRNAs can also significantly influence tumor progression by regulating gene transcription and posttranscriptional modifications [[16], [17], [18]]. Furthermore, research has revealed that tumor drug resistance is closely associated with lncRNAs and with the interactions between immune cells and tumor cells within specific pathways [[19], [20], [21], [22]]. In addition, one prognostic model effectively forecasts the immune profile of individuals with colorectal cancer using four ferroptosis-associated lncRNAs [23]. Therefore, it is highly practical to explore the mechanisms by which cuproptosis-associated lncRNAs (CuALs) regulate tumor immunity and ICI resistance in ESCA.

A close correlation exists between the OS of individuals with ESCA and the accumulation of copper ions. Additionally, lncRNAs are potential biomarkers for identifying patients with an activated immune status. Consequently, it is hypothesized that CuALs could help identify ESCA patients with improved prognostic outcomes and immunotherapy sensitivity. In our study, we identified *UGDH* antisense RNA 1 (*UGDH-AS1*) as a CuAL, which has been studied in SARS-CoV-2 infection and found to be associated with host immune response regulation [24]. Therefore, we hypothesized that *UGDH-AS1* plays a role in ESCA progression. This research evaluates the impact of *UGDH-AS1* on ESCA patient outcomes, aiming to find novel targets for prognostic assessment and therapeutic intervention.

A prognostic signature was developed in this research using three CuALs. This model was formulated by employing Cox regression analysis and the application of the least absolute shrinkage and selection operator (LASSO) method. The signature has been shown to effectively forecast patient prognosis and identify potential recipients who will benefit from ICI treatment. In addition, a nomogram incorporating risk scores and additional clinical characteristics was created. Wound healing experiments confirmed the inhibitory effect of *UGDH-AS1* on the progression of ESCA.

## 2 Materials and methods

### 2.1 Data acquisition

The transcriptomic profiles, somatic mutation information, and relevant clinical data of ESCA patients were downloaded from the Cancer Genome Atlas (TCGA) database (https://portal.gdc.cancer.gov/) and the Gene Expression Omnibus (GEO) database (https://www.ncbi.nlm.nih.gov/geo/). In this investigation, a comprehensive analysis was performed on 291 specimens. These included 161 cancerous and 11 non-cancerous tissue samples sourced from the TCGA database, along with 119 specimens from the GEO cohort (GSE53624). After that, the clinical information of each sample was combined with gene expression data. Samples that had a poor correlation with prognosis were excluded from the TCGA cohort, and 160 samples with complete OS data were obtained. From prior studies, 38 genes associated with cuproptosis were identified (Supplementary Table S1) [[25], [26], [27], [28]]. Furthermore, utilizing Pearson's correlation analysis through the application of the “limma” and “ggplot2” R packages, we successfully identified 442 CuALs (Supplementary Table S2). This identification was determined by a correlation threshold greater than 0.4 and a significance level less than 0.05 [29]. Additionally, TMB in ESCA was evaluated using somatic mutation information from the TCGA database.

### 2.2 Clustering analysis of CuALs

The association between CuAL expression and ESCA subtypes was evaluated using the “ConsensusClusterPlus” R package [30]. Subsequently, all samples were categorized into C1 and C2 according to their CuAL expression levels. This approach improved the correlation within subgroups and reduced it between subgroups. The “Survminer” R package was performed to analyze and contrast the survival rates of the two subgroups.

### 2.3 Construction and verification of the CuAL signature

ESCA samples derived from the TCGA functioned as a training set to develop a prognostic signature. Conversely, samples from the GSE53624 dataset were employed as a validation set. Using univariate Cox regression analysis, four significant CuALs were identified. A signature for predicting prognosis was then developed using LASSO and multivariate Cox regression analysis, resulting in the determination of risk scores for ESCA patients. These scores were then used to classify patients as high- or low-risk groups. Survival differences between these groups were analyzed via the “survminer” package. Time-dependent ROC curves, generated using the “timeROC” R package, assessed the signature's efficacy in forecasting prognosis through AUC values [31]. Multivariate Cox regression further confirmed the independence of the signature. R package “rms” was used to create the nomogram, and the calibration curves validated its clinical applicability.

Patients were stratified using the “ConsensusClusterPlus” R package based on the expression of total genes, cuproptosis-associated genes, 442 CuALs, and 3 CuALs [32]. Principal component analysis (PCA) was conducted to visualize patient group distributions, and the “pheatmap” R package created a heatmap of risk scores and clinicopathological features [33].

### 2.4 Analysis of functional enrichment in immune-related lncRNAs with differential expression

The immune score information of the TCGA samples was downloaded from ESTIMATE (ESTIMATE: Disease (mdanderson.org)). Then, all samples were classified into two groups: one with high immune scores and one with low immune scores, using the median immune score as a threshold. The differentially expressed genes (DEGs) between different immune score groups, as well as those between high- and low-risk groups, were obtained using the “limma” and “edgeR” R packages. By intersecting these DEGs, 29 immune-associated differentially expressed genes (IDEGs) (Supplementary Table S3) were obtained and used for subsequent functional enrichment. Based on these IDEGs, functional enrichment analysis was performed using the “ggplot2” and “clusterProfiler” R packages. In addition, at the genomic level, gene set enrichment analysis (GSEA) has been used to evaluate biological signatures [34]. Therefore, we used the R packages “ggplot2” and “clusterProfiler” for GSEA to determine the enrichment of signaling pathways for these IDEGs.

### 2.5 Tumor-immune landscape analysis

A variety of algorithms, including TIMER, CIBERSORT, QUANTISEQ, MCPCOUNTER, XCELL, and EPIC, were employed to determine the infiltration ratio of immune cells in R software. Subsequently, single-sample gene set enrichment analysis was utilized to investigate the enrichment of these IDEGs in immune-related processes. The “limma” and “ggplot2” R packages were used to determine the relationship between risk score and immune checkpoint gene expression. A box plot was then generated to visually represent this expression pattern.

### 2.6 Tumor mutation burden analysis

Somatic mutations in ESCA patients were analyzed using the R package “maftools”, and a waterfall was also constructed [35]. Furthermore, the “survminer” R package was used to compare the OS differences between the H- and L-TMB groups.

### 2.7 Cell culture and transfection

Cells for this study were obtained from the Cell Bank of the Shanghai Institutes of Biological Science, Chinese Academy of Science (Shanghai, China). Normal esophageal epithelial cell lines (Het-1A) and ESCA cell lines (TE-1 and ECA109) were maintained in DMEM and RPMI 1640 medium, respectively. To provide essential nutrients and growth factors required for cellular growth, fetal bovine serum was added to all culture media. Additionally, the inclusion of penicillin-streptomycin solution was critical for maintaining a sterile environment in the culture medium. The environmental parameters for cell culture were 37 °C, 5 % CO_2_, and 95 % humidity. All reagents used for cell culture were purchased from Gibco, CA, USA.

*UGDH-AS1* was temporarily silenced using a combination of siRNA duplexes and Lipofectamine 3000, following the guidelines provided by the manufacturer (Invitrogen; Thermo Fisher Scientific, Inc., USA). The siRNA sequences can be found in Supplementary Table S4.

### 2.8 RT-qPCR analysis

Cells were collected when the cell density reached 80 %. All subsequent RNA extraction procedures were performed under cold conditions. The pipette tips and centrifuge tubes used were treated to be free of acid, DNase, and RNase. RNA was extracted from cells using the TRIzol method (Invitrogen, MA, USA), and cDNA synthesis was conducted using a PrimeScriptTM RT reagent kit (TaKaRa, Kyoto, Japan). Reverse transcription conditions were set at 42 °C for 45 min, and then the reverse transcriptase was inactivated by heating at 85 °C for 5 min. Subsequently, synthesized cDNA was analyzed using RT-qPCR with the SYBR Green MasterMinds Kit on the Biosystem 7500 Testing System Platform (ABL, USA) according to the provided guidelines. The primers utilized can be found in Supplementary Table S5.

### 2.9 Wound healing assay

The cells from both the *UGDH-AS1* siRNA group and the control group were initially harvested from the culture dishes, resuspended in PBS, and centrifuged to eliminate any remaining trypsin. Subsequently, cells were resuspended and counted. The cells were then seeded at a density of 1 × 10^5^ cells per well in a 24-well plate and incubated for 24 h. Once the cell density reached 80 %, they were transferred to a 6-well plate. After incubating for an additional 12 h to ensure full adhesion, we applied a consistent scratch to the surface of the adherent cells. PBS was then used to wash away any cells dislodged by this process. Observations of cell migration and wound healing were conducted using light microscopy at the 0-h and 24-h time points.

### 2.10 Statistical analysis

The R packages used included “ConsensusClusterPlus”, “survminer”, “pheatmap”, “glmnet”, “timeROC”, “scatterplot3d”, “org.Hs.eg.db”, “ggplot2”, “enrichplot”, “clusterProfiler”, “ggpubr”, “limma”, and “maftools”. Statistical significance was defined as a P value < 0.05. Finally, to evaluate the significance of the statistical results derived from the study, a one-way ANOVA was employed. All statistical analyses were performed using R (version 4.2.1) and GraphPad (version 8.0) software. *P < 0.05, **P < 0.01, ***P < 0.001.

## 3 Results

### 3.1 Identification of CuALs associated with ESCA prognosis

The general design and procedure are illustrated in Fig. 1, which was created with BioRender.com. Gene expression profiles and clinical data for patients with ESCA were retrieved from the TCGA database and the GSE53624 dataset. The TCGA cohort served as the primary source for the development of the prognostic signature and acted as a training set. Within this set, the signature was initially constructed and refined. Meanwhile, the GSE53624 dataset served as a test set to verify the effectiveness of the prognostic signature in different patient populations. In the TCGA cohort, we identified 442 CuALs by Pearson's correlation analysis (Fig. 2A). Finally, data from 160 ESCA patients was obtained after the exclusion of 12 patients with incomplete clinical information.

**Fig. 1:**
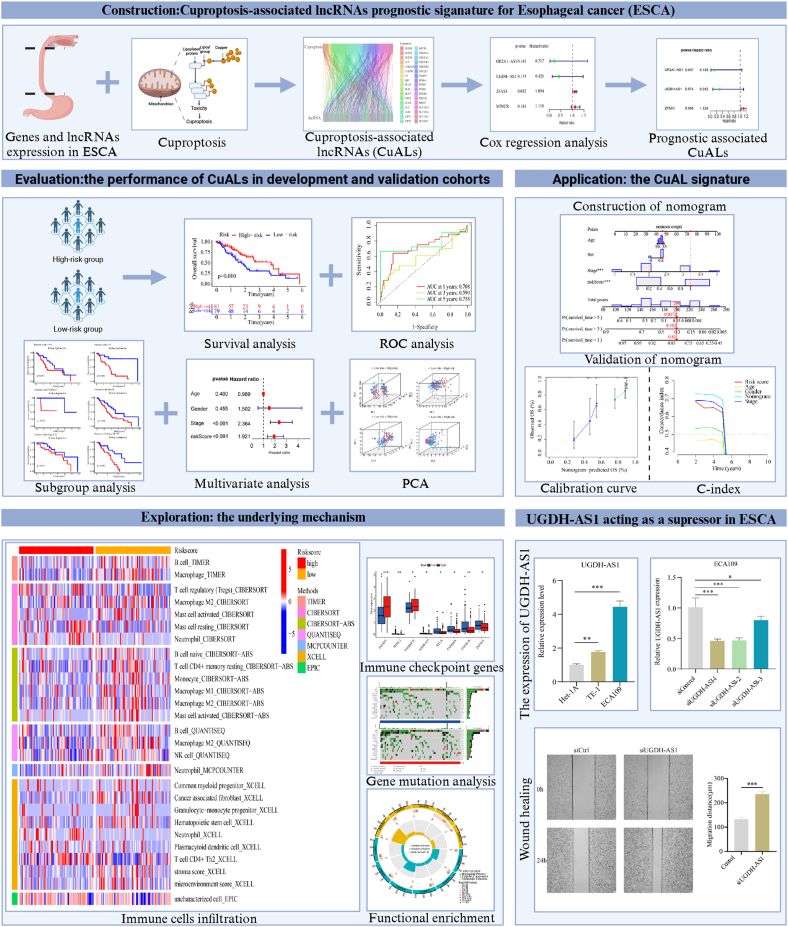
General schematization of the study.

**Fig. 2:**
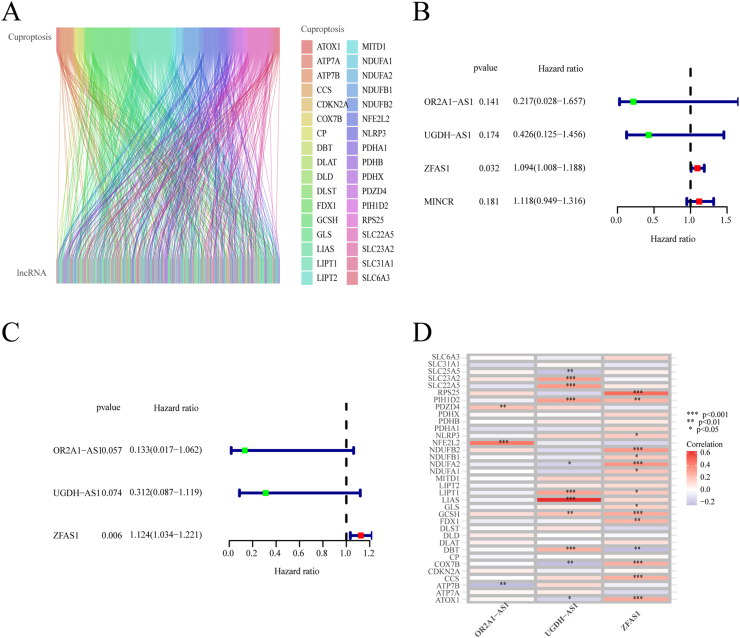
Construction of the prognostic signature for ESCA. (A) Sankey diagram depicting the co-expression network of cuproptosis-associated genes and CuALs. (B) Univariate Cox analysis of four CuALs. (C) Multivariate Cox analysis of three CuALs. (D) Correlation analysis heatmap between the cuproptosis-associated genes and the three CuALs. *P < 0.05, **P < 0.01, ***P < 0.001.

### 3.2 Characteristics of cuproptosis clusters in ESCA

For a more comprehensive understanding of the significance of the 442 CuALs, samples from the TCGA cohort were stratified into two categories, labeled C1 and C2. This classification was based on the differential expression patterns observed among these CuALs (Supplementary Figs. S1A–C). In addition, the difference in OS between C1 and C2 was validated using K-M survival analysis. Notably, patients with ESCA in the C2 subgroup exhibited a substantially extended OS than those in subgroup C1 (P < 0.01) (Supplementary Fig. S1D). Heatmap showing the differences in gene expression and clinical features between C1 and C2. Interestingly, C1 showed higher expression levels of CuALs, while there were no differences in clinical factors (Supplementary Fig. S1E). These results demonstrate that the 442 CuALs are correlated with the prognosis for ESCA patients. Consequently, these CuALs have the potential to be utilized in stratifying ESCA patients into different prognostic groups and thus providing more individualized treatment.

### 3.3 Establishment of the CuAL prognostic signature within the ESCA patient group

Within the training group, four CuALs were found to have a significant correlation with survival rates through univariate Cox regression analysis. Forest plots were also generated (Fig. 2B). Subsequently, a prognostic signature composed of three lncRNAs, *OR2A1* antisense RNA 1 (*OR2A1-AS1*), *UGDH* antisense RNA 1 (*UGDH-AS1*), and *ZNFX1* antisense RNA 1 (*ZFAS1*), was developed through the application of both LASSO Cox regression and multivariate Cox regression analysis, and the risk score of each sample was also obtained (Fig. 2C). As shown in the corrplot, there was a significant correlation between these three CuALs and cuproptosis-associated genes (Fig. 2D). Genes with a hazard ratio <1, such as *OR2A1-AS1* and *UGDH-AS1*, were considered tumor suppressors, while *ZFAS1*, which has a hazard ratio >1, was considered an oncogene.

### 3.4 Validation of the CuAL prognostic signature

**Fig. 3:**
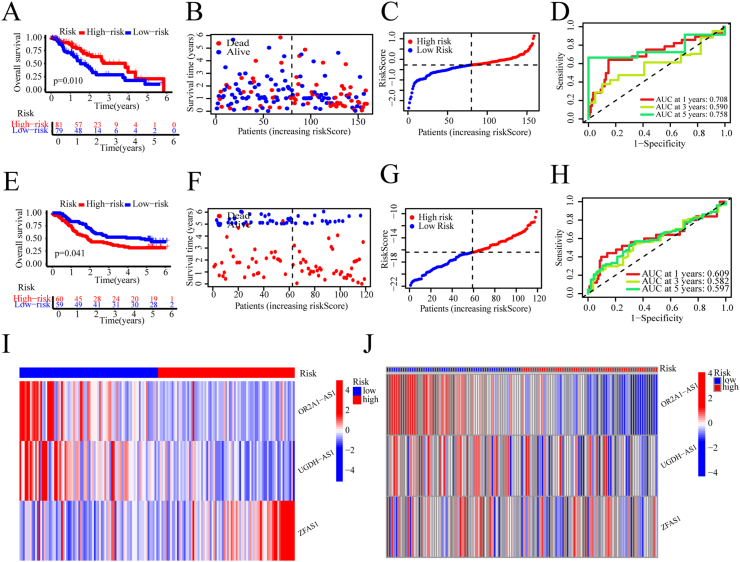
Validation of the three CuALs prognostic signature. (A–D) The overall survival (A), survival status distribution (B), risk score curves (C), and ROC curves (D) of the training group. (E–H) The overall survival (E), survival status distribution (F), risk score curves (G), and ROC curves (H) of the test cohort. (I and J) Expressions and distributions of 3 CuALs in training (I) and test cohorts (J). Statistical significance was defined as a P value < 0.05.

Patients with ESCA were classified as either high- or low-risk based on the median risk score. K-M survival analysis revealed that individuals within the high-risk group experienced a markedly lower survival rate (p < 0.001) (Fig. 3A and E). Furthermore, the survival status distribution plot of the prognostic signature revealed a corresponding decline in OS for ESCA patients as the risk score increased (Fig. 3B and F). The arrangement of samples and median value of the risk score could also be found (Fig. 3C and G). This prognostic signature demonstrates enhanced predictive accuracy, as evidenced by AUC values of 0.708, 0.590, and 0.758 for 1-, 3-, and 5-year forecasts, respectively (Fig. 3D). Comparable results were observed in the GSE53624 test cohort, confirming the signature's reliability across different datasets (Fig. 3H). Furthermore, as depicted in Fig. 3I and J, individuals in the high-risk group exhibited lower *OR2A1-AS1* and *UGDH-AS1* expression and higher *ZFAS1* expression. Lower expression of *OR2A1-AS1* and *UGDH-AS1* was associated with shorter OS in ESCA patients, while *ZFAS1* showed the opposite trend (Fig. 9A, Supplementary Fig. S2 E and F). This result is consistent with the above conclusion that *OR2A1-AS1* and *UGDH-AS1* are tumor suppressors, while *ZFAS1* is an oncogene.

The prognostic signature employed PCA to distinguish between high- and low-risk groups and to evaluate their gene expression profiles. The analysis included the expression of total genes, 38 cuproptosis-associated genes, 442 CuALs, and 3 CuALs (Supplementary Figs. S2A–D). The PCA results indicate that the prognostic signature is effective in differentiating between different risk groups.

### 3.5 The prognostic signature serves as an independent variable

**Fig. 4:**
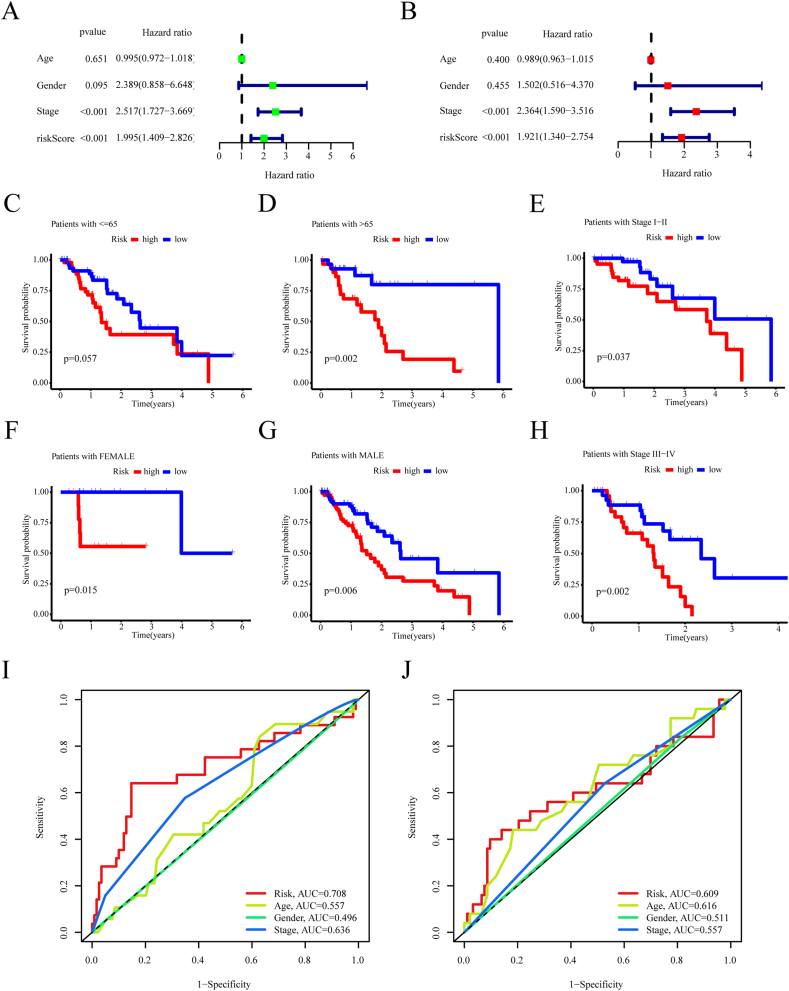
The predictive value of the prognostic signature. (A and B) Analysis of clinicopathological factors and risk scores using multivariate Cox regression analysis for the training cohort (A) and the test cohort (B). (C–H) K-M curves were performed on patients with different risk scores within various clinicopathological factor subgroups: Age (C and D); Stage (E and H); and Sex (F and G). (I and J) The AUC values associated with risk score and other prognostic factors in the training (I) and test cohorts (J). Statistical significance was defined as a P value < 0.05.

Given that patient prognosis is often correlated with several clinical variables, we examined the ability of this prognostic signature to independently forecast patient outcomes, apart from other clinical characteristics, through multivariate Cox analyses within the TCGA and GSE53624 cohorts. In both cohorts, this prognostic signature was found to have a significantly independent value in predicting patient outcomes (Fig. 4A and B). Furthermore, except for patients aged 65 years or younger, ESCA patients in the high-risk group had significantly decreased survival rates compared with those in the low-risk group according to sex (FEMALE, p < 0.05; MALE, p < 0.01) and pathological stage (stage I-II, p < 0.05; stage III-IV, p < 0.01) (Fig. 4C–H), suggesting that the prognostic signature is a valuable tool for predicting ESCA patient outcomes in different clinical feature groups.

The prognostic signature demonstrated strong sensitivity and specificity in the prediction of ESCA patient outcomes, as evidenced by time-dependent ROC analysis. Overall, the signature outperformed traditional prognostic factors such as age (AUC = 0.557), sex (AUC = 0.496), and pathological stage (AUC = 0.636) in predicting OS, highlighting its significant predictive power (Fig. 4I). Comparable results were also found in the GSE53624 test cohort (Fig. 4J).

### 3.6 Construction and validation of the nomogram

**Fig. 5:**
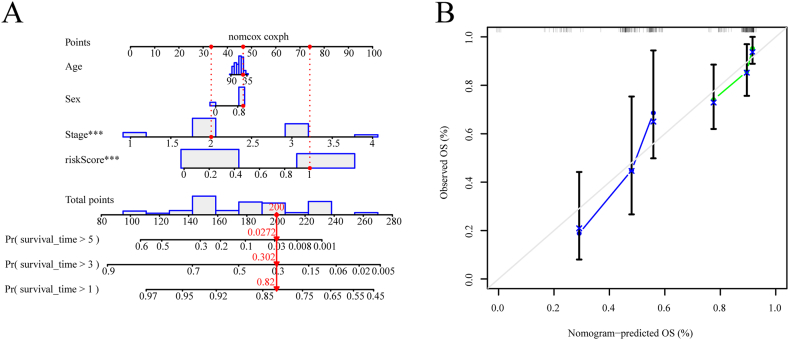
Construction and accuracy validation of the nomogram. (A) A nomogram was constructed to predict ESCA patients' survival. (B) Calibration curves for evaluating nomogram's efficacy. *P < 0.05, **P < 0.01, ***P < 0.001.

A nomogram incorporating the risk score and other traditional prognostic features was constructed to expand the use of prognostic signatures for ESCA. This model enabled more comprehensive prediction of 1-, 3-, and 5-year survival probabilities for patients, thereby improving the accuracy of the prognostic signature used in clinical practice (Fig. 5A). The nomogram indicated that the prognostic signature, constructed using three CuALs, had the greatest impact on OS. The calibration curve exhibited a strong relationship between forecasted and actual OS at 1- and 3-year, but not at 5-year (Fig. 5B). In addition, the nomogram model had a greater C-index than any of the other risk factors (Supplementary Fig. S2G), indicating the favorable predictive power of the nomogram model. Overall, the findings suggest that incorporating multiple factors into a prognostic signature, such as a nomogram utilizing a risk score and clinicopathological features, leads to improved predictive accuracy.

### 3.7 Immune-associated differentially expressed genes functional enrichment analysis

**Fig. 6:**
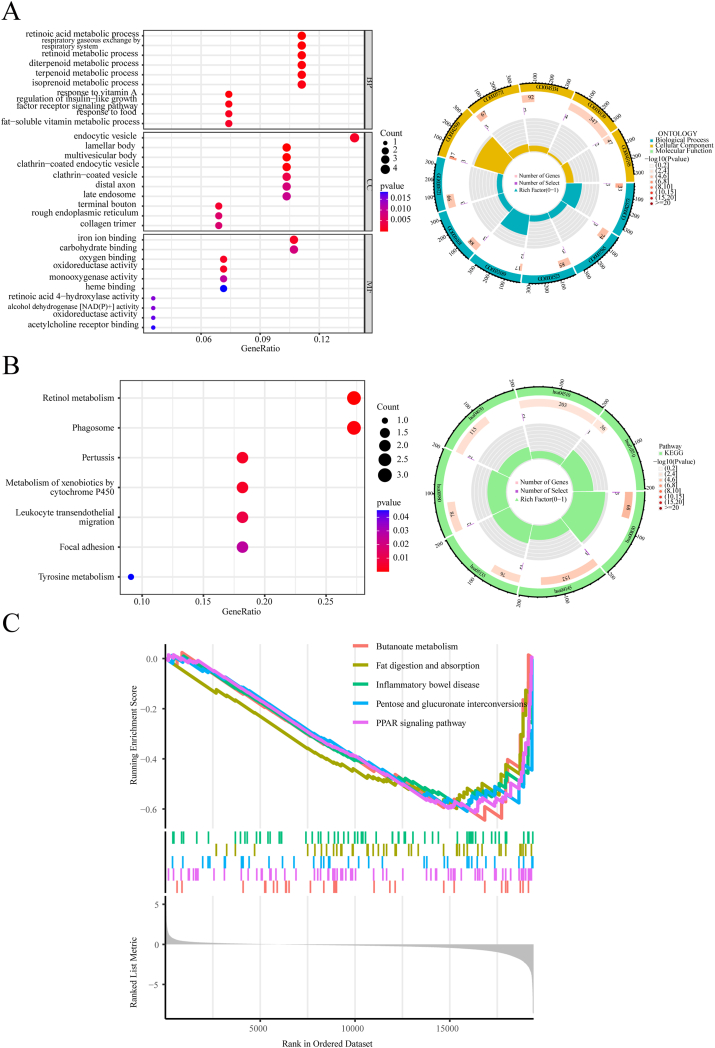
Analysis of IDEGs for functional enrichment. (A) Bubble graphs and circle diagram of IDEGs for GO enrichment. (B) Bubble graphs and circle diagrams of IDEGs for KEGG pathway enrichment. (C) GSEA pathway enrichment analysis for IDEGs.

Immune scores were obtained from the ESTIMATE database for patients with ESCA within the TCGA cohort. To elucidate the correlation between patients’ immune status and tumor outcomes, DEGs were identified between two different risk groups as well as between two groups with different immune scores. We then intersected these DEGs. This led to the discovery of 29 IDEGs. Within the biological process category, GO analysis indicated that these IDEGs are predominantly concentrated in pathways associated with vitamin and insulin, such as retinoic acid metabolism and the retinoid metabolic process, in response to vitamin A. Cellular components were associated with endocytic vesicles, lamellar bodies, and multivesicular bodies. The molecular functions identified through GO annotation included iron ion binding and carbohydrate binding, among others (Fig. 6A).

Furthermore, the IDEGs were associated with various pathways, including focal adhesion, phagosomes, and leukocyte transendothelial migration, as indicated by the KEGG analysis (Fig. 6B). This suggests that IDEGs may influence the immune response in ESCA patients. Additionally, to further elucidate the distinctions in biological pathways between the two risk groups, GSEA was employed. This analysis revealed that downregulated gene sets are primarily enriched in 18 cancer-associated pathways, including the PPAR signaling pathway, pentose and glucuronate interconversions, inflammatory bowel disease, and other pathways (Fig. 6C). Therefore, the IDEGs derived from the CuAL prognostic model, alongside high- and low-immune scores, are predominantly linked to tumor immunity. This indicates the potential for further investigation into the immune differences in patients with different risk scores.

### 3.8 Immune landscape of risk scores in patients with ESCA

**Fig. 7:**
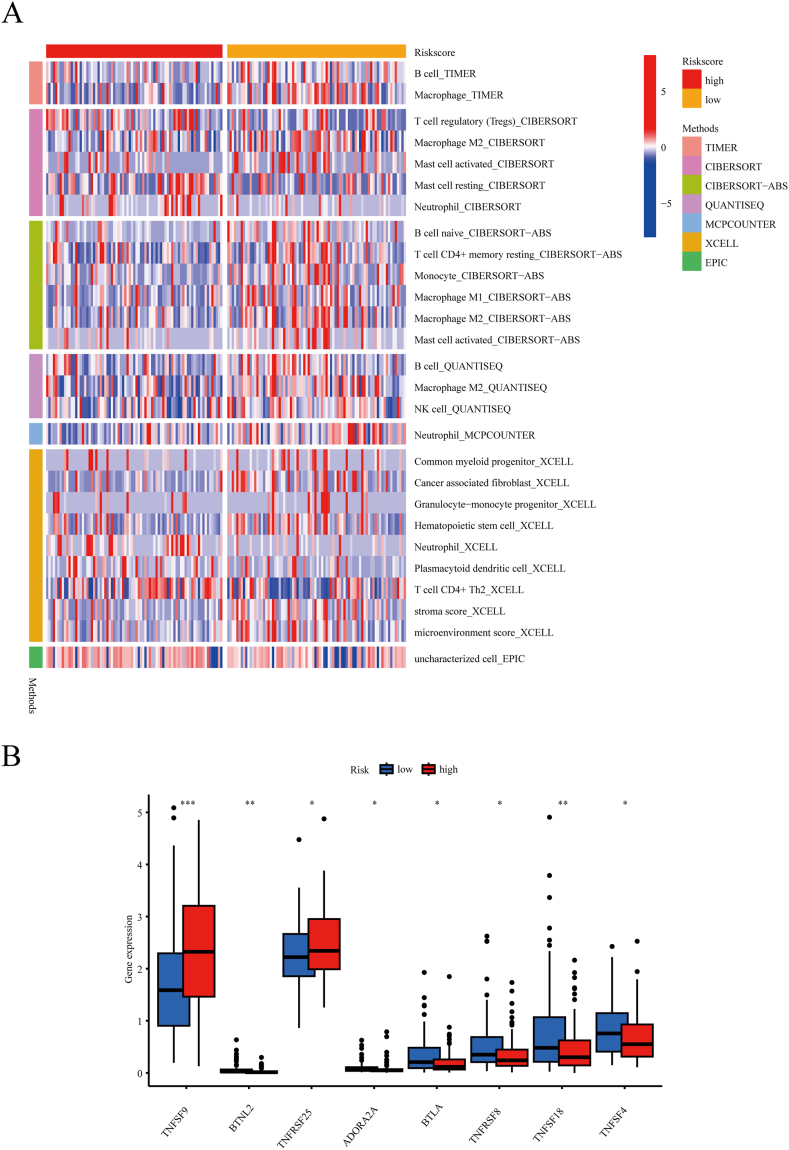
Immune landscape of the CuAL signature in ESCA patients. (A) The distribution of tumor-infiltrating immune cells between the high-risk and low-risk groups. (B) ESCA patients' box plots of immune checkpoint genes differentially expressed by risk groups. *P < 0.05, **P < 0.01, ***P < 0.001.

The tumor microenvironment is constituted by a variety of cell types, which include tumor cells, immune cells, stromal cells, and so on [38]. Nontumor cells, especially immune cells, are essential in the modulation of tumor progression and metastasis. To investigate the association between the prognostic signature and tumor immunity in ESCA, several algorithms, including TIMER, CIBERSORT, and QUANTISEQ, were employed to assess immune cell infiltration in patients with ESCA. Patients classified as low-risk exhibited increased infiltration of T cells and macrophages, among others (Fig. 7A). In contrast, patients identified as high-risk showed limited immune cell infiltration, primarily of mast cells and neutrophils.

ICIs have become one of the most effective treatments for ESCA. Identifying potential recipients is essential for improving the effectiveness of immunotherapy in ESCA. Therefore, we examined the differences in immune checkpoint gene expression levels between the high-risk and low-risk groups to assess the potential utility of the prognostic signature in identifying beneficiaries. The findings suggested that the low-risk group had higher levels of immune checkpoint gene expression compared to the high-risk group. Significantly elevated levels of *BTLA*, *TNFRSF8*, *TNFSF18*, and *TNFSF4* were observed in low-risk patients, while *TNFSF9* and *TNFRSF25* showed the opposite trend (Fig. 7B). Therefore, the low-risk group exhibited increased expression of immune checkpoint genes and more immune cell infiltration compared to the high-risk group. The result showed potentially favorable outcomes for low-risk patients treated with ICIs. This suggests that the prognostic signature may be effectively utilized to discern which patients are susceptible to immunotherapy.

### 3.9 Genomic mutation analysis for prognostic signatures

**Fig. 8:**
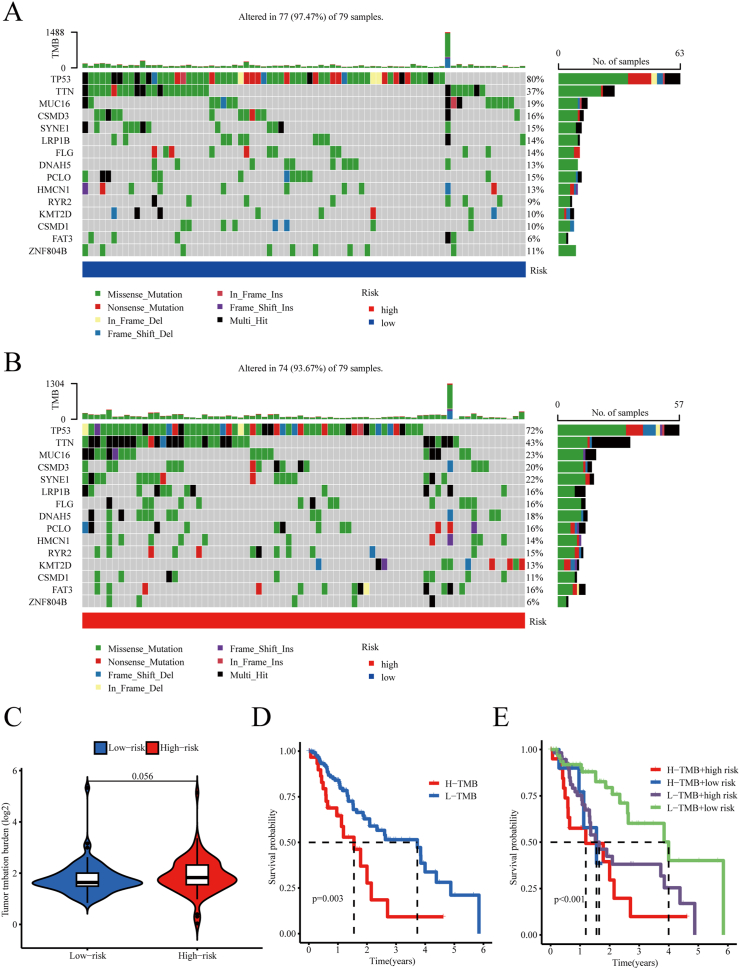
Genomic mutation analysis for prognostic signatures. (A and B) The waterfall plots of somatic gene mutations in low- (A) and high-risk groups (B), respectively. (C) TMB comparison of two groups. (D) K-M curves for OS between the two groups. (E) K-M curves for OS among four different groups. Statistical significance was defined as a P value < 0.05.

To examine the influence of somatic mutations on patient outcomes in ESCA, mutation data was extracted from the TCGA database, and the TMB was calculated. Within the low-risk group, the most frequently mutated genes were *TP53* (80 %), *TTN* (37 %), *MUC16* (19 %), *CSMD3* (16 %), and *SYNE1* (15 %) (Fig. 8A). Conversely, within the high-risk group, *TP53* (72 %), *TTN* (43 %), *MUC16* (23 %), *CSMD3* (20 %), and *SYNE1* (22 %) were the most frequently mutated genes (Fig. 8B).

**Fig. 9:**
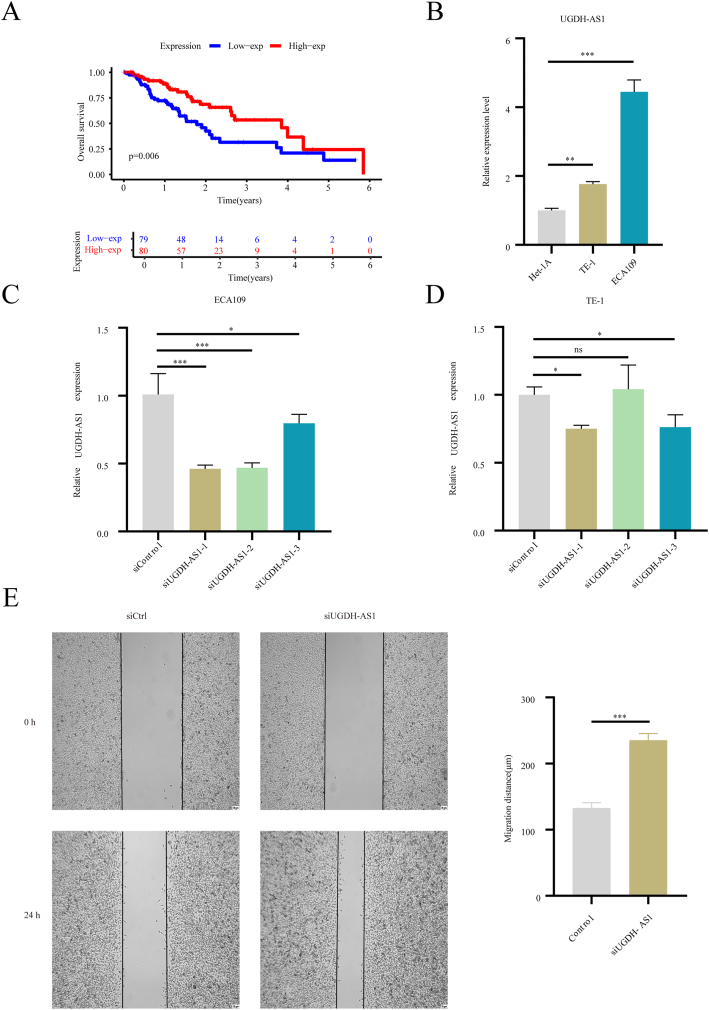
*UGDH-AS1* suppressed the migration of ESCA. (A) K-M curves for OS between differentially expressed *UGDH-AS1* groups. (B) *UGDH-AS1* upregulated in ESCA cell lines. (C and D) Expression of *UGDH-AS1* after transfection with interference reagents in ECA109 (C) and TE-1 (D) cell lines. (E) Wound healing assay and corresponding statistical analysis. ns, not significant; *P < 0.05, **P < 0.01, ***P < 0.001.

Furthermore, to explore the impact of TMB on OS, samples were then stratified into groups with H- or L-TMB based on the median TMB score as a threshold. Although the TMB discrepancy between the two risk groups was not prominent, the patients in the L-TMB group showed a more favorable survival rate (p = 0.003) (Fig. 8C and D). Furthermore, within the subgroups, individuals with both H-TMB and high risk scores had the poorest prognosis, whereas those with L-TMB and low risk scores had a more favorable survival outcome (P < 0.001) (Fig. 8E). Therefore, we hypothesize that the TMB is also a powerful prognostic tool for ESCA.

### 3.10 Identification of potential targets of cuproptosis

The above findings underscore the significant clinical predictive value of the CuAL prognostic signature. Subsequently, we examined the impact of *UGDH-AS1*, *OR2A1-AS1*, and *ZFAS1* on patient survival. Although both *UGDH-AS1* and *OR2A1-AS1* acted as suppressors of ESCA, *UGDH-AS1* exhibited greater prognostic efficacy and a higher correlation with cuproptosis-associated genes than *OR2A1-AS1* (Figs. 2D and 9A, and Supplementary Fig. S2E). In addition, patients with higher expression of *ZFAS1* exhibited a low survival rate compared to those with low expression (Supplementary Fig. S2F). Given that most existing studies have focused primarily on exploring the functions of oncogenes, we selected *UGDH-AS1* as a target for further study. *UGDH-AS1* was upregulated in the ESCA cell lines TE-1 and ECA109, as demonstrated by RT-qPCR analysis (Fig. 9B). Furthermore, interference with *UGDH-AS1* (Fig. 9C and D) increased the metastatic ability of ESCA cells (Fig. 9E). These findings demonstrated that *UGDH-AS1* is overexpressed in ESCA and is negatively correlated with ESCA progression.

## 4 Discussion

ESCA is recognized as the sixth-leading cause of cancer-related deaths in the world [1]. Although some treatment options, such as surgery, chemotherapy, radiotherapy and immunotherapy, show some benefit in clinical care, outcomes for ESCA patients remain unsatisfactory [5,36]. This may be associated with the lack of precise methods for predicting patient prognosis and immune landscapes, which hinders the provision of precisely tailored individual treatments. Currently, commonly used factors to predict the prognosis of ESCA include the histopathological stage of the tumor, along with patient sex and age. However, the use of these factors for predictions is limited in practice [36,37]. Therefore, more reliable and accurate prognostic signatures are urgently needed for ESCA patients. Molecular typing of tumors offers a new approach to exploring the heterogeneity of ESCA, assessing prognosis, and recommending personalized treatment options. Therefore, establishing a novel prognostic signature by molecular typing of ESCA is critically essential to enable personalized treatments and enhance OS for patients with this disease.

Cuproptosis, an unusual copper-dependent cell death mechanism, was reported by Tsvetkov et al. [12]. This pathway differs significantly from the well-characterized processes of apoptosis and necrosis. Copper ions, typically serving as cofactors, are essential in modulating biological functions. Under normal circumstances, these ions facilitate mitochondrial respiration and energy metabolism through their interaction with enzymes and proteins [38]. However, if the balance of copper ions is disrupted, it can lead to various pathological conditions associated with copper dysregulation, such as oxidative stress and abnormal autophagy. Additionally, lncRNAs are becoming more widely acknowledged as essential regulators in the development of cancer. They modulate gene transcription and post-transcriptional modifications [17,18]. The utility of lncRNAs has evolved beyond their traditional biological functions; recent advances have harnessed their potential to develop prognostic signatures. These signatures, based on the predictive capabilities of lncRNAs, have proven highly effective in identifying potential populations that would benefit from ICI treatment [39]. Furthermore, prognostic signatures based on CuALs have been extensively researched. These models hold significant importance for predicting clinical outcomes across various cancers. For example, in gliomas and lung adenocarcinomas, these prognostic models have achieved AUC values of 0.749 and 0.756, respectively [40,41]. These findings highlight the potential of CuALs as powerful biomarkers for predicting patient prognosis and treatment efficacy. Given the proven efficacy of CuALs in these cancers, we hypothesized that similar prognostic models could be equally beneficial for patients with ESCA.

This research introduced a novel prognostic signature for ESCA comprising three CuALs (*OR2A1-AS1*, *UGDH-AS1*, and *ZFAS1*). In the TCGA cohort, the signature was developed using LASSO and multivariate Cox regression analysis. It was then externally validated in the GSE53624 dataset, confirming its efficacy across different study populations. In this context, *OR2A1-AS1* and *UGDH-AS1* may act as suppressors in ESCA, as their decreased expression correlates strongly with unfavorable patient outcomes. Conversely, *ZFAS1* may function as an oncogene, given that its expression patterns and prognostic impacts are opposite to those of the aforementioned lncRNAs. As evidenced by the results, individuals with elevated risk scores in ESCA exhibited decreased levels of *OR2A1-AS1* and *UGDH-AS1* and increased levels of *ZFAS1*. Prognostic analysis revealed that patients with elevated risk scores, whether from the TCGA cohort or the GSE53624 dataset, exhibited significantly reduced OS. These results underscored the prognostic signature's broad applicability in accurately forecasting patient outcomes across multiple populations. Furthermore, the prognostic signature of CuALs has been confirmed to have the capability to predict patient prognosis independently of other clinical factors, as demonstrated by multivariate Cox regression analyses. The prognostic signature demonstrates considerable predictive accuracy, evident from the elevated AUC values. However, it is acknowledged that prognostic predictions based on a single biomarker have limitations in predicting patient survival [42]. To address this, a nomogram was established that integrates the risk score with various clinicopathological factors to enhance the accuracy of patient outcome predictions. The nomogram demonstrates high consistency between the predicted and observed OS, with a C-index surpassing that of other risk factors. Thus, it provides a more reliable tool for clinical prognosis and decision-making in the management of ESCA. This approach could not only refine the precision of medical assessment. It could also facilitate the tailoring of personalized treatment strategies.

In this study, differentially expressed genes analysis was conducted among patients stratified into various risk and immune score groups, leading to the identification of 29 IDEGs. Subsequently, the impact of these IDEGs on the tumor immune environment was further investigated. GO and KEGG functional enrichment analyses indicated a strong correlation between the IDEGs and biological processes, including isoprenoid metabolism, endocytic vesicles, iron ion binding, and retinol metabolism. Notably, isoprenoids have been shown to regulate T cells by suppressing activated Treg cells [43]. The PPAR pathway, which is one of the most enriched pathways according to GSEA, may exert an influence on the growth and apoptosis of colorectal cancer cells [44]. Overall, IDEGs appear to regulate biological processes within both cancer and noncancer cells in the TME. On the other hand, the prognostic signature could facilitate the early identification of patients who are optimal candidates for immunotherapy, potentially enhancing therapeutic outcomes and improving survival rates.

Considering that copper ions can participate in various physiological activities, they may also affect the TME of ESCA. Previous research has demonstrated that an increase in copper ions within tumors can upregulate PD-L1 expression, leading to tumor immune escape. However, this effect is significantly inhibited upon the introduction of copper ion chelators [45]. Consequently, an analysis was conducted to evaluate variations in immune checkpoint gene expression and immune cell infiltration among patients between two different risk groups. The findings revealed that individuals in the low-risk group exhibited decreased *TNFSF9* and *BTNL2* expression, as well as enhanced infiltration of resting memory CD4^+^ T cells, and so on. However, individuals with a high risk score exhibited the opposite trend, which is consistent with their poor prognosis. This suggests that the suppression of the immune system may contribute to the reduced OS observed in high-risk patients. These results highlight the immunological differences that correlate with different prognostic risk groups. In recent research, it was found that two immune checkpoint genes, *TNFSF9* and *TNFRSF25*, were upregulated in the high-risk category, while six other genes were downregulated. Research indicated that *TNFRSF25* signaling has a positive impact on the proliferation of CD_4_^+^ lymphocytes by increasing the production of IL-2RA, IL-2RB, and IL-2 [46]. Conversely, *BTLA*, which is present in most lymphocytes, acted as an immune suppressor by inhibiting B and T cells from proliferating and activating [47]. Previous research has indicated a strong correlation between activated NK cells and a favorable prognosis for individuals with cancer [48]. Our research showed reduced infiltration of NK cells in high-risk individuals with unfavorable outcomes. This is in line with earlier findings. In conclusion, there is a significant difference in immune checkpoint gene expression and infiltration of immune cells between different risk groups. This distinction may influence patients' responses to immunotherapy. Additionally, these findings highlight the prognostic signature's capacity to differentiate patients with varying immune landscapes.

TMB is frequently used as a prognostic indicator for the efficacy of immunotherapy in individuals with cancer. Our research revealed that patients classified as high-risk with an unfavorable prognosis had more mutations in the *TTN* and *MUC16* genes. Previous research has indicated a correlation between mutations in *TTN* and increased responsiveness to ICI therapy in solid tumors, while *MUC16* mutations are linked to cancer advancement and prognosis. In this study, patients with high TMB exhibited decreased OS, thereby underscoring the impact of TMB on the prognosis of ESCA. Given that both the risk score and TMB have demonstrated utility in prognostic prediction, we integrated the patient risk score with the TMB for further classification to enhance the predictive accuracy. The results showed more precise prognostic predictions, indicating that a multifactorial consideration in prognosis prediction leads to increased accuracy.

In this study, we observed a pronounced upregulation of *UGDH-AS1* gene expression in ESCA cell lines, which demonstrated a significant correlation with the disease's prognosis. Previous research has reported that the upregulation of *UGDH-AS1* is linked to decreased disease severity through the modulation of *MOV10* and *EDN1* [49]. In the present investigation, it was observed that interference with *UGDH-AS1* expression markedly improved the migration ability of cancer cells. This finding supports the conclusion that *UGDH-AS1* acts as a protective factor in tumor progression. For the other genes in the prognostic signature, *OR2A1‐AS1* was discovered to have decreased expression in individuals with diffuse large B-cell lymphoma and was linked to a reduced OS [50]. This underscores the potential role of *OR2A1-AS1* as a biomarker for poor prognosis in cancer. Furthermore, recent studies have highlighted the role of the *IMP2*-*ZFAS1*-*OLA1* signaling pathway in modulating mitochondrial energy metabolism during colorectal cancer progression [51].

However, there are still some limitations to our study. For instance, the information on ESCA was obtained from public datasets that require further validation at multiple centers. Additionally, among the genes comprising this prognostic signature, only *UGDH-AS1* has been studied. A further study is needed to investigate *OR2A1-AS1* and *ZFAS1* roles in carcinogenesis.

## 5 Conclusions

Overall, the prognostic signature utilizing three CuALs demonstrated high accuracy and sensitivity when it came to forecasting an ESCA patient's prognosis. Furthermore, this signature was able to identify individuals who may respond well to ICIs. According to the CuAL prognostic signature, ESCA patients with a low risk score demonstrated a significantly prolonged OS. Furthermore, these patients exhibited an immune-active phenotype, suggesting potential efficacy from treatment with ICIs. In addition, *UGDH-AS1*, a CuAL, was negatively correlated with ESCA progression. The findings of this research established a foundational framework for further exploration of prognostic diagnosis and immunotherapy in ESCA.

## Ethics approval and consent to participate

Not available.

## Data availability statement

This study utilized publicly accessible datasets for analysis.

## Funding

This study was supported by Joint Funds for the Innovation of Science and Technology, Fujian Province (Grant Number: 2021Y9057).

## CRediT authorship contribution statement

**Xinhai Sun:** Writing – review & editing, Writing – original draft, Visualization, Validation, Resources, Investigation, Formal analysis, Data curation, Conceptualization. **Liming Li:** Writing – review & editing, Writing – original draft, Validation, Resources, Data curation. **Xiaojie Yang:** Writing – original draft, Investigation, Data curation, Conceptualization. **Dan Ke:** Resources, Investigation, Formal analysis. **Qihong Zhong:** Writing – review & editing. **Yuanchang Zhu:** Resources, Investigation, Formal analysis. **Litao Yang:** Resources, Investigation, Formal analysis. **Zhenyang Zhang:** Writing – review & editing, Supervision, Project administration, Investigation, Funding acquisition, Conceptualization. **Jiangbo Lin:** Writing – review & editing, Writing – original draft, Supervision, Project administration, Investigation, Funding acquisition, Conceptualization.

## Declaration of competing interest

The authors declare that they have no known competing financial interests or personal relationships that could have appeared to influence the work reported in this paper.

## Figures and Tables

**figs1:**
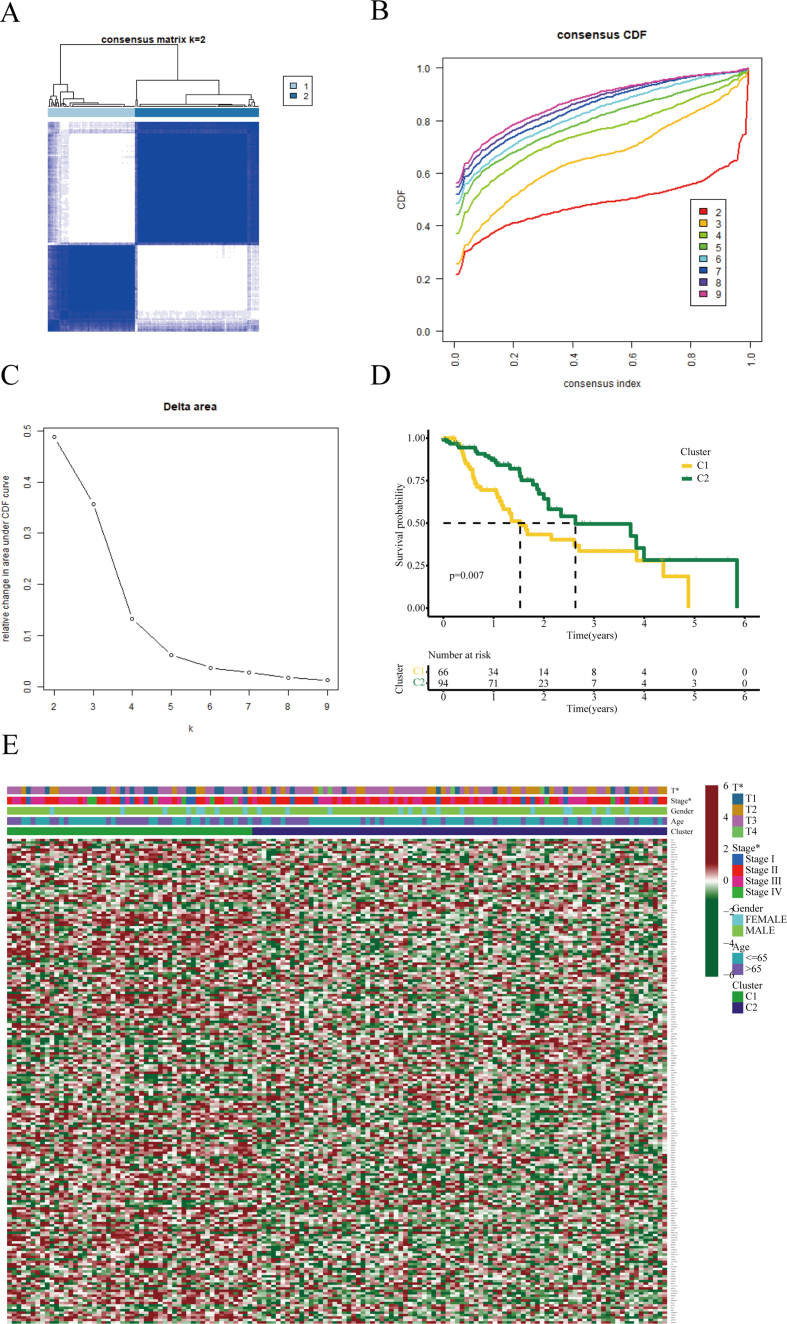


**figs2:**